# Dr. Henry Skelton

**Published:** 1913-12

**Authors:** 


### DR. HENRY SKELTON.

HENRY SKELTON, M.D. St. And., died on December 8th, after a short illness, at the age of 71, having given forty-four years of strenuous medical work to a large area surrounding his residence at Downend.

He held many medical appointments in Downend, Frenchay, Hambrook, Keynsham, Mangotsfield, Stapleton and Warmley. He was a man of wide sympathies and considerable ability, and was accordingly much respected as a very popular practitioner of medicine and surgery. He was devoted to the church and to music. A Freemason and a Conservative politician, he was also a member of many committees embracing a large number of useful objects, and did some good work in training nurses for the British Red Cross Society.

"A man he was to all the country dear."

He will be greatly missed by the members of the many societies for which he worked, and by his *clientele* of patients, in whom he inspired great confidence.

